# The Development of a Universal *In Silico* Predictor of Protein-Protein Interactions

**DOI:** 10.1371/journal.pone.0065587

**Published:** 2013-05-31

**Authors:** Guilherme T. Valente, Marcio L. Acencio, Cesar Martins, Ney Lemke

**Affiliations:** 1 Department of Morphology, UNESP – Univ Estadual Paulista, Botucatu, Sao Paulo, Brazil; 2 Department of Physics and Biophysics, UNESP – Univ Estadual Paulista, Botucatu, Sao Paulo, Brazil; Semmelweis University, Hungary

## Abstract

Protein-protein interactions (PPIs) are essential for understanding the function of biological systems and have been characterized using a vast array of experimental techniques. These techniques detect only a small proportion of all PPIs and are labor intensive and time consuming. Therefore, the development of computational methods capable of predicting PPIs accelerates the pace of discovery of new interactions. This paper reports a machine learning-based prediction model, the Universal *In Silico* Predictor of Protein-Protein Interactions (UNISPPI), which is a decision tree model that can reliably predict PPIs for all species (including proteins from parasite-host associations) using only 20 combinations of amino acids frequencies from interacting and non-interacting proteins as learning features. UNISPPI was able to correctly classify 79.4% and 72.6% of experimentally supported interactions and non-interacting protein pairs, respectively, from an independent test set. Moreover, UNISPPI suggests that the frequencies of the amino acids asparagine, cysteine and isoleucine are important features for distinguishing between interacting and non-interacting protein pairs. We envisage that UNISPPI can be a useful tool for prioritizing interactions for experimental validation.

## Introduction

Graph or network theory has been used to model complex systems such as social and biological aspects [1], [2], and provides a good interface between the reductionist and holistic views [3]. In biological networks, the nodes represent biological elements (e.g. biomolecules) and links or edges represent physical or functional interactions among these biological elements [3]. These networks have shown to be useful for deciphering the behavior of biological systems and their computational inference can sharply reduce the costs of experimental identification of novel interactions [1], [3] with a high applicability to drug targets discovery, for example [3]. Usually, the predictions are based on similarity-based algorithms, maximum likelihood methods or probabilistic methods; the typical applications of those algorithms are concerning the reconstruction of networks, evaluation of network evolving mechanism, and classification of partially labeled networks [1]. In the molecular networks, the links can be predicted based on the node features (e.g. protein sequences or domains), by the similarities of edge neighborhood, by comparisons to an appropriated model, by network topology, and so on [3].

Proteins are one of the most abundant classes of biomolecules that can interact with many other biomolecules in cells, such as DNA, RNA, metabolites and other proteins. The latter interactions – protein-protein interactions (PPIs) – are essential interactions that build functional units responsible for the functioning of all biological molecular pathways [4]. Consequently, building a list of all of an organism's PPIs can be useful for the molecular-level understanding of the core of complex traits. Therefore, the collection of all PPIs can be important for understanding the underlying mechanisms of diseases, facilitating the process of drug design, elucidating the functions of newly identified proteins, predicting their subcellular location and gaining insight into the evolution of some interaction or metabolic pathways, among other biological aspects of a cell or organism.

Physical PPIs can be identified using experimental methods, such as the yeast two-hybrid assay, mass spectrometry, protein microarrays, phage display, X-ray crystallography, fluorescence resonance energy transfer, surface plasmon resonance, atomic force microscopy and electron microscopy [5]. However, these experiments, in addition to being expensive and time demanding, are not suitable for all proteins and do not report all interactions that can occur in cells or organisms [5]–[7]. Therefore, a computational approach capable of reliably predicting PPIs can identify the potential interactions to be further interrogated using experimental approaches.

Several computational methods for predicting physical or functional protein interactions based on the information from several experimental and computational approaches, such as phylogenetic profile analysis, gene co-expression profiles, sequence co-evolution, synthetic lethality data, *in silico* two-hybrid systems, gene cluster and gene neighbor analysis, protein domain information [7], [8], protein interface analysis, protein docking methods [9], [10], and orthology and ontology information [11], among others, have been published. Moreover, several data sources can be used as examples for training several machine learning (ML) algorithms in methods of classification [8], [12]–[16]. Additionally, it is possible to combine more than one approach and dataset to predict PPIs [8], [17]–[20]. However, some of the aforementioned methods use datasets that are not available for all species or all proteins. For instance, some methods use information from complete genome sequencing, gene-expression data or protein information that is not available for all sequences. Moreover, some methods have flaws: gene expression can not determine physical PPIs; molecular evolutionary events can disturb the prediction of PPIs in phylogenetic profile methods; functional relationships based on genomic organization are only suitable for prokaryotes; some methods are based on sequence homology, which is more difficult to determine than finding genes; and other computational and biological aspects may render the prediction very difficult or do not provide resolute results. Some of these points are argued in some papers [7], [21], [22].

The primary protein structure, does not considering domains, motifs, ontology, phylogenetic trees and orthology, has sufficient information to estimate the propensity for PPIs [3], [23]. Moreover, amino acid (aa) sequences are the most universal protein feature and thus appear to be ideal traits for use in building methods of predicting PPIs that are applicable to all proteins [24]. In fact, many interesting and useful bioinformatics methods using primary sequences have been developed, and many methods include machine learning approaches [12], [15], [16], [24]–[36]. However, some of the methods based on ML and primary sequences have weaknesses, such as the building of negative datasets, the small number of examples and the large number of attributes (vectors) that code protein pairs.

To overcome these limitations, we present in this paper the Universal *In Silico* Predictor of Protein-Protein Interactions (UNISPPI), an ML-based approach that use features associated with amino acid sequences for building models for predicting PPIs. UNISSPI is a probabilistic model and presents the following advantages over previously mentioned methods: (1) the negative training examples - the non-interacting protein pairs (hereafter named no-PPIs) - used to construct the training datasets are all experimentally supported no-PPIs; (2) the amount and diversity of PPIs and no-PPIs used as training examples are much higher than some of those used by other methods; (3) its computational cost is markedly reduced in comparison to other methods because of the small amount of learning features used to generate the models (only 20 features); and (4) the generated models are decision trees that can be easily analyzed and the most important features capable of distinguishing PPIs from no-PPIs can be promptly identified. By only considering 20 attributes associated with amino acid sequences as learning features, UNISPPI can correctly classify 79.4% and 72.6% of known PPIs and no-PPIs, respectively, including protein pairs from eukaryotes, prokaryotes, viruses, and parasite-host associations. Because of these characteristics, we consider UNISPPI a truly universal predictor that is appropriate for large-scale prediction and thus can become a useful tool for indicating the most relevant interactions, which can be further validated experimentally.

## Results

### Predictive performance of generated models

**Figure 1:**
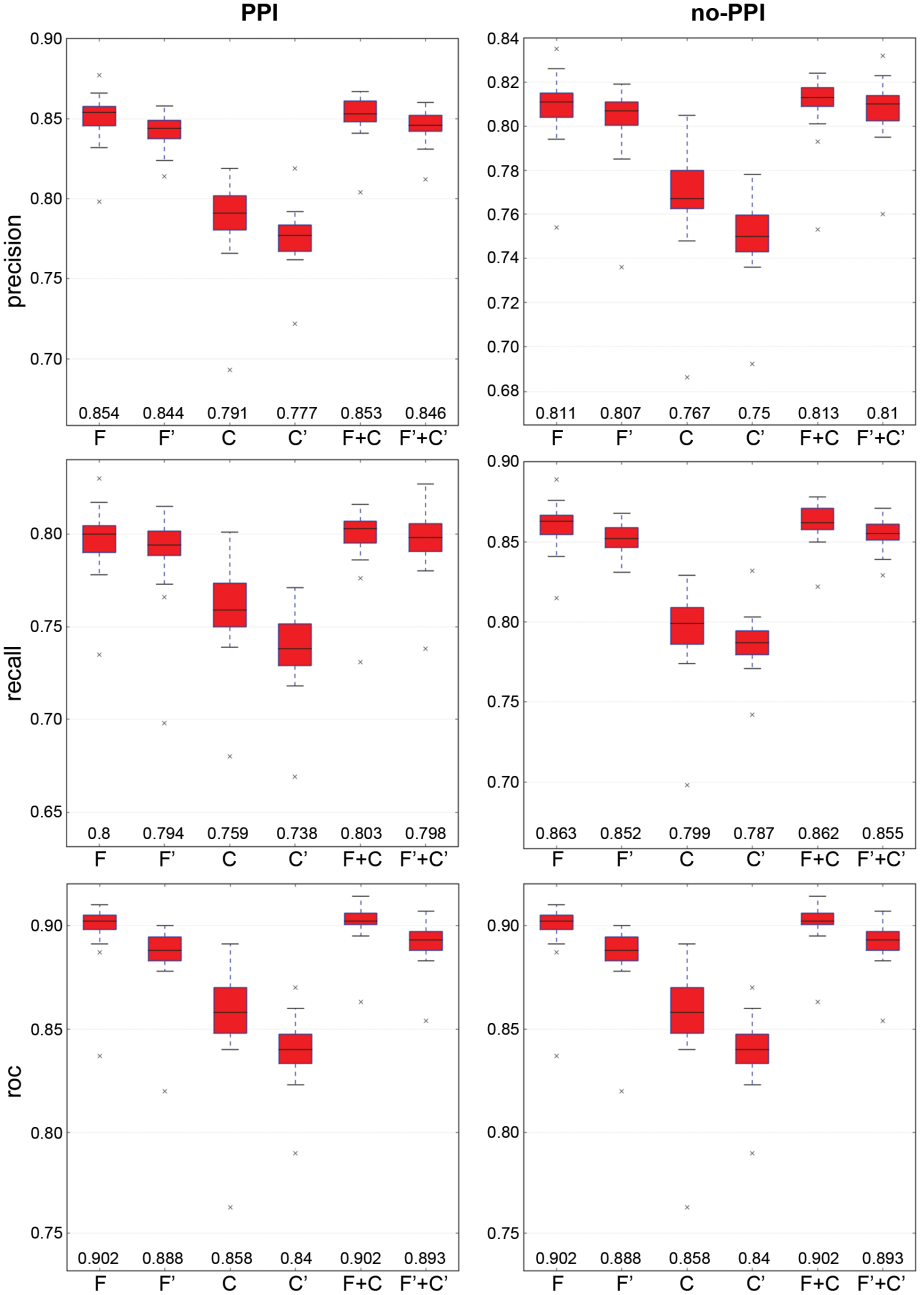
Predictive performance of the machine learning using the Normal training datasets. The letters F, C and F+C indicate that the Normal training datasets originated from the feature descriptors “frequency”, “composition” and “frequency” plus “composition”, respectively. The prime symbol indicates the Normal training datasets formed using the symmetrical attributes of the previously mentioned datasets (details in the “Material and Methods” section). The numbers at the bottom of the boxes are the medians for each dataset.

The predictive performance of prediction models – in this case, decision tree models – generated from the Normal and Random training datasets are reported in the Figure 1 and Figure S1, respectively. The predictive performances of the decision tree models generated from different groups of the Normal training datasets appear to be slightly different from each other (Figure 1), but it is evident that from their performance that models generated from the C or C′ datasets resulted in the lowest predictive values. Interestingly, the Mann-Whitney U statistical test showed that the performance measures are significantly different for almost all of the performed comparisons (Table 1). For these cases, we assumed that the best set of features that were able to discriminate the PPI and no-PPI classes were those that produced decision tree models with the highest median predictive values. Regarding the models with no significant differences, we assumed that both datasets used to generate the models had the same predictive performance. Thus, as seen in Table 1, the models generated from F and F+C are the most predictive models, followed by those generated from F′ and F′+C′. Moreover, the performance measures for models generated from these four training datasets vary by up to 1.4% (AUC), and the lowest variation is 0.6% (precision values for no-PPIs). Furthermore, the decision tree models created from Random datasets showed performance measures of approximately 0.5, confirming the hypothesis that the Normal datasets are able to extract relevant information about PPIs and no-PPIs (Figure S1).

**Table 1:** Mann-Whitney U statistical test applied to performance measures of models generated from Normal training datasets.

Datasets		PPI	NO-PPI
		p-value	p-value
C×C′	Precision	1.15E-04	2.43E-06
C×C′	Recall	3.51E-06	0.002
C×C′	AUC	1.73E-06	1.73E-06
C×F+C	Precision	1.18E-11	1.61E-10
C×F+C	Recall	5.36E-10	8.36E-12
C×F+C	AUC	2.26E-11	2.26E-11
C×F′	Precision	8.41E-12	5.61E-10
C×F′	Recall	4.38E-09	0.000
C×F′	AUC	1.51E-09	1.51E-09
C×F′+C′	Precision	8.36E-12	1.61E-10
C×F′+C′	Recall	9.90E-10	7.21E-12
C×F′+C′	AUC	1.17E-10	1.17E-10
C′×F+C	Precision	7.54E-12	2.17E-11
C′×F+C	Recall	5.08E-11	0.000
C′×F+C	AUC	7.46E-12	7.46E-12
C′×F′+C′	Precision	7.49E-12	1.49E-11
C′×F′+C′	Recall	2.76E-11	7.56E-12
C′×F′+C′	AUC	8.33E-12	8.33E-12
F+C×F′+C′	Precision	9.00E-04	1.76E-02
F+C×F′+C′	Recall	*9.15E-02	0.002
F+C×F′+C′	AUC	2.46E-08	2.46E-08
F×C	Precision	1.72E-11	2.11E-10
F×C	Recall	7.28E-10	1.11E-11
F×C	AUC	1.40E-10	1.40E-10
F×C′	Precision	7.49E-12	2.18E-11
F×C′	Recall	3.84E-11	0.000
F×C′	AUC	4.59E-11	4.59E-11
F×F+C	Precision	*2.49E-01	*8.91E-02
F×F+C	Recall	*1.27E-01	*2.79E-01
F×F+C	AUC	*1.34E-01	*1.34E-01
F×F′	Precision	6.91E-04	1.61E-02
F×F′	Recall	*7.23E-02	0.001
F×F′	AUC	4.62E-08	4.62E-08
F×F′+C′	Precision	1.25E-02	*1.97E-01
F×F′+C′	Recall	*4.14E-01	1.61E-02
F×F′+C′	AUC	4.67E-06	4.67E-06
F′×C′	Precision	7.54E-12	1.11E-10
F′×C′	Recall	1.40E-10	0.000
F′×C′	AUC	1.16E-10	1.16E-10
F′×F+C	Precision	3.14E-05	2.31E-04
F′×F+C	Recall	2.86E-03	1.50E-04
F′×F+C	AUC	4.57E-10	4.57E-10
F′×F′+C′	Precision	*9.50E-02	*9.49E-02
F′×F′+C′	Recall	*9.75E-02	*0.162
F′×F′+C′	AUC	1.12E-02	1.12E-02

* , no significant difference.

### Analysis of decision tree models

Beyond their prediction capability, decision tree models can be used for knowledge acquisition to describe patterns in datasets. Decision trees are decision support tools inferred from the training data that use a graph of conditions and their possible consequences. The structure of a decision tree consists of a root node representing the most important condition for discriminating classes and internal nodes representing additional conditions for class discrimination under the main condition [37].

**Figure 2:**
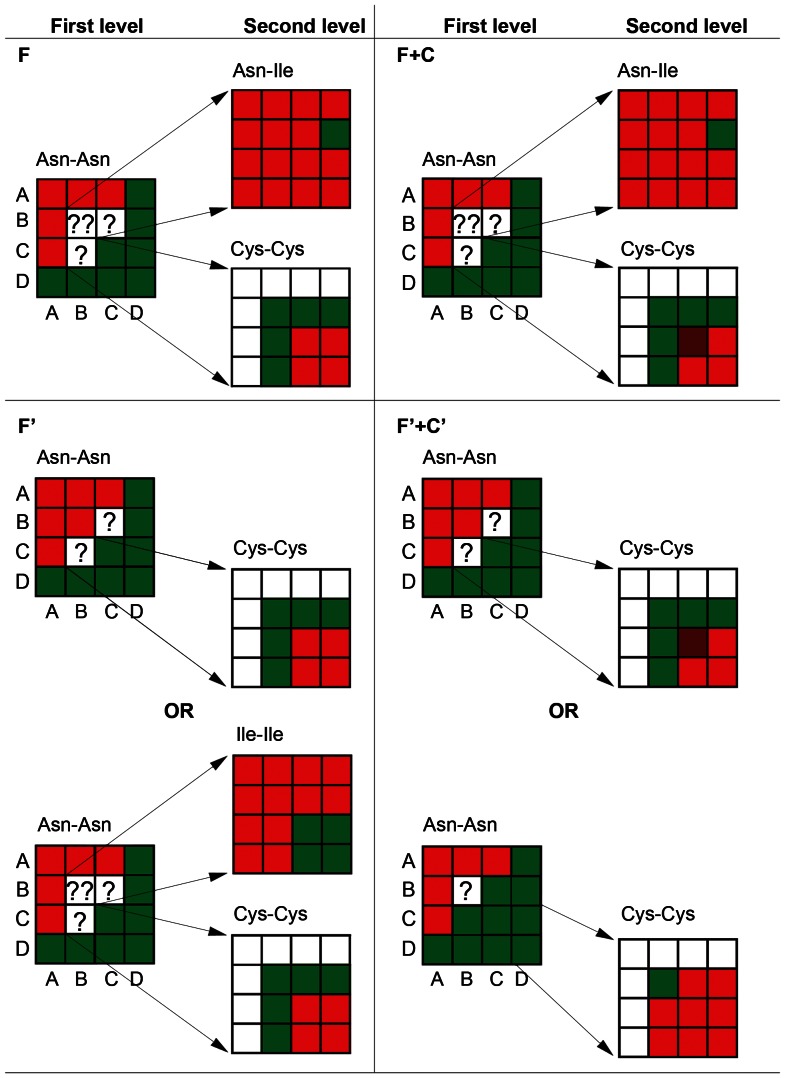
Synthesis of several decision trees generated during the ML training. The aa at the top of each box is relative to the attributes specified at the first and second level of the trees. A, B, C and D indicate the low, moderate, high and very high bins, respectively (for more details, see Table 3 and the “Materials and Methods” section). Green boxes, a combination that classifies an instance as a PPI; Red boxes, a combination that classifies an instance as a no-PPI; Brown boxes, a combination that classifies an instance as a PPI or no-PPI; “?”, a combination for which an instance can not be classified, requiring classification at the next level of the tree.

Of the 124 decision tree models generated by training the J48 algorithm on the F, F′, F+C and F′+C′ Normal training datasets (31 models for each dataset) (see “Materials and Methods” for details), we analyzed 24 decision trees (six for each dataset). The decision trees show that the combination frequencies of asparagine in the proteins from a protein pair (Asn-Asn) is the root node of the decision trees generated from all four datasets. Thus, this attribute can be considered the most important feature for discriminating the PPIs and no-PPIs of a dataset. Interestingly, some frequencies of Asn are not able to discriminate PPIs from no-PPIs (22 from all analyzed trees); in these cases, the combination frequencies of cysteines (Cys-Cys) or asparagine with cysteine (Asn-Cys) or asparagine with isoleucine (Asn-Ile) or isoleucines (Ile-Ile) in the proteins from a protein pair are relevant features for classifying the instances (Figure 2; Table S1). Cysteine comprises 54.4% of the amino acids in the second level of the analyzed trees, whereas Ile and Asn comprise 26.5% and 19.1%, respectively, at the same level in all analyzed trees.

### Classification of unlabeled instances

**Figure 3:**
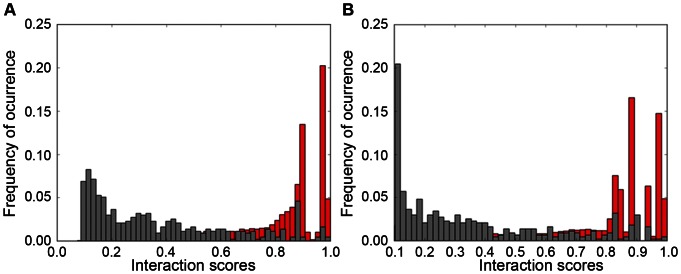
Graphics of classification of the test sets using the F and F′ Normal combined models. The score represents the classification of the instances as PPI. The instances were classified as PPIs or no-PPIs, and no-PPIs classification scores were converted to interaction scores. “A”, classification of the F test set using the F Normal combined model; “B”, classification of the F′ test set using the F′ Normal combined model; Red, PPIs instances; Gray, no-PPIs instances.

**Figure 4:**
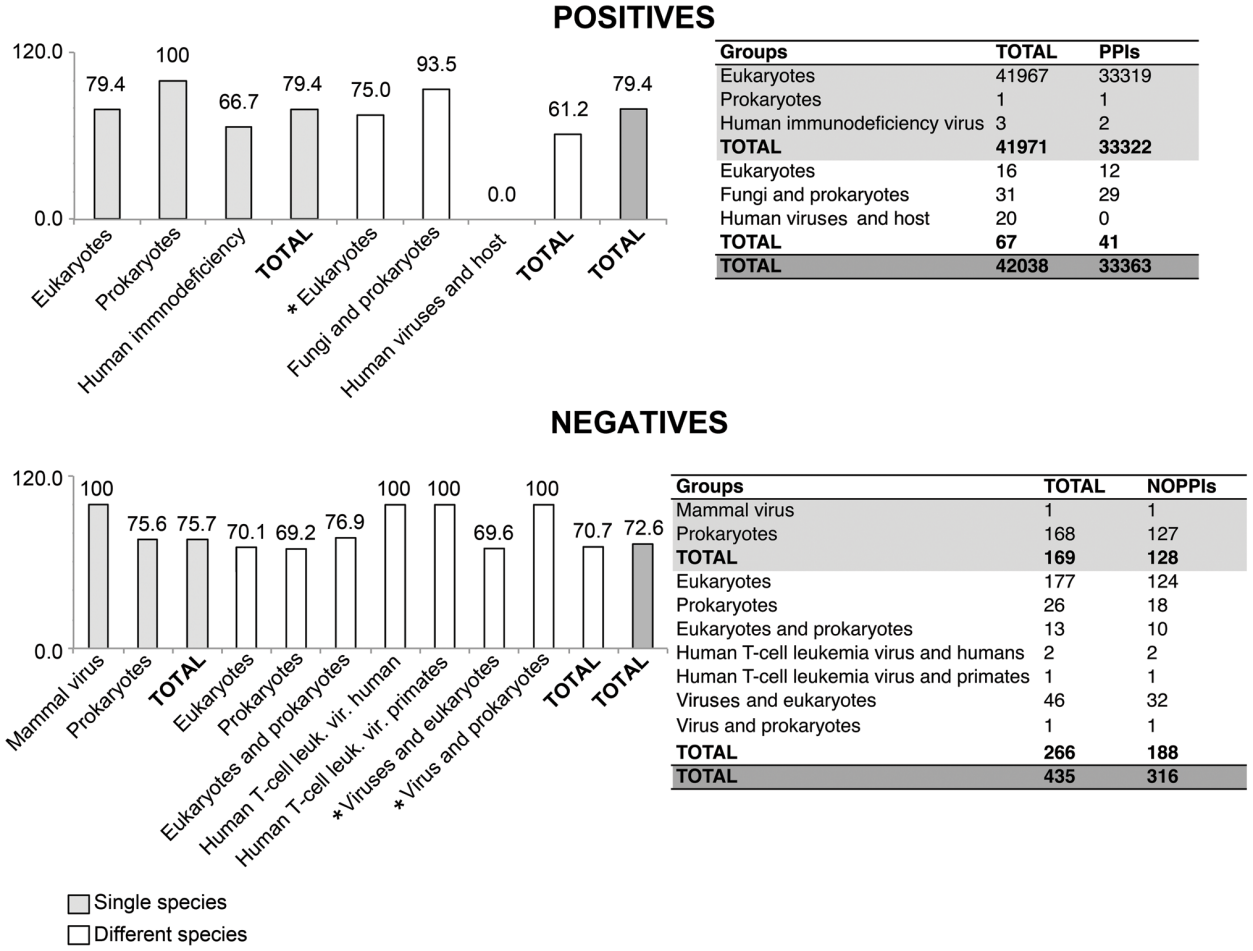
Detailed analysis of the classification of the test set using the F′ Normal combined model. The values in the right side of each table and the score over each bar indicate the number and percentages of instances correctly classified, respectively. Single species, indicates PPIs and no-PPIs within the same species; Different species, indicates PPIs and no-PPIs among different species; *, groups that include instances of parasite-host associations.

The combined models generated from the F and F′ Normal datasets (see “Materials and Methods” for details) were used independently to classify the test set (Figure 3). In general, both the F and F′ Normal combined model are able to correctly classify ∼79.5% and ∼73% of PPIs and no-PPIs, respectively (Table 2). A more detailed analysis of the test set classified by the F′ Normal combined model revealed that this set of features was able to classify species-specific PPIs and no-PPIs for prokaryotes, eukaryotes and viruses and PPIs and no-PPIs among different species (also including proteins from eukaryotes, prokaryotes and viruses). Some PPIs and no-PPIs from parasite-host associations were also correctly predicted (for instance, protein interactions between *Plasmodium falciparum* and humans; between proteins of the human immunodeficiency virus, Epstein-Barr virus, influenza A virus, human cytomegalovirus, simian virus, chimpanzee hepatitis and human hosts; between the Sendai virus and mouse hosts; and between an enterobacteria phage and *Escherichia coli* host). Moreover, some no-PPIs among virus proteins and a non-host species were also correctly classified (Figure 4; Table S2).

**Table 2:** Summary of the number of instances of the test set correctly classified using the F and F′ Normal combined models.

	Classification using F Normal combined model		Classification using F′ Normal combined model	
Classes	numb. of instances	% of instances	numb. of instances	% of instances
PPI	33,609	79.9	33,363	79.4
no-PPI	319	73.3	316	72.6

numb., number.

Moreover, the F and F′ Normal training sets were described as unlabeled instances and were also classified using the F and F′ Normal combined models, respectively. The results indicated that in general the instances were classified correctly (Figure S2). Moreover, the F and F′ Random combined models were applied to classify both the test set and the training set (in this case, described as unlabeled instances) separately, and the models were not able to classify those unlabeled instances (Figure S3).

## Discussion

In this paper, we presented a new method called Universal *In Silico* Predictor of Protein-Protein Interactions (UNISPPI), based on primary sequence information and a small set of features, for classifying protein pairs as interacting or non-interacting proteins. To build the final UNISPPI, a large number of tests were conducted, and several protein physicochemical features were examined. The following discussion will show how and why we chose the core set of features for this method.

### Selecting the best physiochemical features to form the UNISPPI and evaluating the universality of this method

Using an ML method, the ability of “frequency” and “composition” physicochemical features in predicting PPI and no-PPI was assessed. The performance measures of decision tree models constructed from six Normal datasets (F, F′, C, C′, F+C and F′+C′) showed that “composition” features do not provide the most relevant information for discriminating PPIs and no-PPIs in a dataset. However, the “frequency” features appear to have the most relevant information for predicting PPIs and no-PPIs. We are not arguing that the “composition” features are not important to PPIs; however, these features do not provide the most relevant information for discriminating a PPI based on our method. Regardless, the statistical analysis indicated that the F and F+C datasets generated the models with the best predictive performances, followed by F′ and F′+C′. However, the variation of the predictive values observed for these Normal datasets, in practical terms, do not differ expressively.

The use of decision trees was very helpful not just to extract information from the datasets but also to generate trees that yielded interesting results. The trees revealed that the combination of the frequencies of Asn, Cys and Ile were the most important feature for classifying the PPIs and no-PPIs. The frequency of Asn in symmetrical attributes (the frequency of Asn on both proteins) was the most important feature, even when the “composition” features were present. The Cys, Ile and Asn frequencies were present at the second level of the tree, in which symmetrical attributes containing Cys (the frequency of Cys on both proteins) were commonly reported. In conclusion, these trees lead us to conclude that the composition does not appear to convey relevant information for predicting PPIs and no-PPIs according to all analyzed trees, corroborating the conclusions obtained from analyzing ML predictive performances.

For the biological role of those three amino acids in PPIs, we considered several reports that showed the relationship between their frequency and PPIs. Analyzing the preference index of inter-chain contacts showed that the interactions between two Asn (Asn-Asn), two Cys (Cys-Cys), between Cys with other residues (including Ile), two Ile (Ile-Ile), and between Ile with other residues, have high preference index values (Cys-Cys presents the highest values) [38].

Regarding the interface features, there are interesting studies showing the participation of Asn, Cys and Ile in protein interfaces. Asn is one of the aa residues identified as having an affinity for interfaces [39], [40] and is present in the binding sites of antibody complementarity-determining regions [41], [42], for example. Moreover, Asn can play a role in stabilizing the protein structure of solvent-accessible regions of ubiquitin-associated domains, which might represent a binding surface [43]. Ile and other hydrophobic and charged residues constitute 25% of the overall number of residues at protein-protein interfaces [38]. Finally, Cys bridges are present in interfaces more frequently than expected [44]. Interestingly, Cys residues are able to make disulfide bonds, and these bonds are common between two Cys residues and are important in cofactor binding, inter-subunit interactions, DNA binding inhibition, membrane binding, subcellular localizations, stabilizing interactions and protein structures [45].

Concerning the test of the models generated by ML training, we used the F and F′ Normal combined models for classifying unlabeled instances of the F and F′ test sets. These models were selected based on the differences in the predictive performances between F and F+C and between F′ and F′+C′, which were not statistically significant. Moreover, the “composition” features do not provide the most relevant information for predicting the interactions. Both models were equally able to classify the unlabeled instances with good predictive performance. These results were very exciting because these models were built using only eukaryotic examples, whereas the test set included a large number of prokaryote, eukaryote and virus examples. Additionally, the results showed that those models are in fact universal. Moreover, the ML training using Random datasets and the classification of the test set using the Random model provided support for all analyses and conclusions for this topic.

Finally, we concluded that using only the frequencies of amino acids in a protein pair was sufficient to classify PPIs and no-PPIs. According to the aforementioned facts, we decided that the best set of features for forming the UNISPPI is the F′ Normal dataset. Although the F Normal dataset is slightly more predictive than the F′ Normal dataset, both models have a similar ability to classify unlabeled protein pairs. Furthermore, the F′ Normal dataset was chosen instead of the F′+C′ Normal dataset because the differences between the predictive performances were not significant and because the decision trees showed that the “composition” information may be irrelevant. Moreover, the dataset chosen (F′) to build the final UNISPPI contains only 20 attributes, which is more desirable than 841, 400 or 41 attributes. In large-scale projects, millions or billions of unlabeled protein pairs must be classified, and, in those cases, the F′ dataset is obviously more useful than all other sets of features. These results support our conclusion that the primary sequence alone can be used to classify a PPI and showed that the predictive performance using only symmetrical attributes was sufficient.

### The innovations of UNISPPI

To show the advantages of UNISPPI over other similar computational methods, we built a chart that showed the features of several similar computational methods (see method descriptions and citations in Table S3). Despite their usefulness, these methods show some limitations that we attempted to overcome through the development of UNISPPI. These limitations mainly encompass the construction of the negative example dataset and the number of instances and attributes in both training and test sets.

For the negative dataset in all other methods, the negative instances were obtained from predictions. These procedures are questionable because, it is easy to introduce bias into the dataset (for training and cross-validation procedures), which may be caused by the over-representation of negative examples and/or by intrinsic aspects of the negative dataset prediction procedure [36], [46]. Moreover, the predictive performances differ according to the negative dataset prediction method used [25], [28], [31], [34]. Some methods rely on *in silico* artificial proteins from the PPI dataset to build the negative examples, and this procedure overestimates the results according to the level of randomness used during the artificial protein building. Moreover, it is intrinsically more difficult to distinguish real protein sequences than artificial ones because artificial sequences can lack specific protein patterns, such as motifs, domains or other signatures. Thus, the models generated using real protein sequences are more reliable and give better results for classifying real protein-protein interactions [28]. In addition, some methods predict that if two proteins are not present as an interacting protein pair within the PPI dataset, those proteins can be included as a non-interacting protein pair to build the negative dataset. This statement does not make sense because it is not known whether these proteins do interact in an organism under some biological circumstances, such as pathogen attack, environmental influences, developmental stages and tissue-specific interactomes, among others (see method descriptions and citations in Table S3).

Some methods are based on a low number of examples or are relevant to just one or few species. Thus, these methods can not be considered universal predictors because of their basis on a small universe of proteins (low diversity). In all methods with well-reported attribute constructions, the number of attributes is usually very high, which renders the methods unfeasible for large-scale prediction procedures. Moreover, some methods were not applied to classify instances that were not in the training set; thus, the true performance of these methods was not tested (see method descriptions and citation in Table S3).

Direct comparisons among the predictive performance of the methods are not possible because of the differences in their features, interactions or methods for developing the negative dataset. Regardless, the UNISPPI has predictive values that are usually higher or comparable to those of the other methods (Table S3).

Finally, UNISPPI is based on primary sequence information that is coded in 20 symmetrical attributes (F′ construction), which is feasible for use in large-scale projects (approximately millions or billions of interactions). Moreover, because the UNISPPI model is based only on experimentally validated examples from diverse species and can be applied to predict PPIs or no-PPIs in a large number of species, the UNISPPI model is truly a universal predictor. This method can easily classify instances of a complete proteome (it builds an interactome) or proteins from parasite-host associations when attempting to reduce the number of interactions to be further investigated using experimental procedures. Since the interactomes are the most promising networks for drug target candidate discovery [3], UNISPPI method can be useful to pace of detection of these targets as well.

To learn how to apply UNISPPI in unlabeled instances, see Text S1. The F′ Normal combined model (the UNISPPI per se) is available in the Dataset S1. Eventually, UNISPPI will return predicted PPIs and a probabilistic score assigned to each interaction.

## Materials and Methods

**Figure 5:**
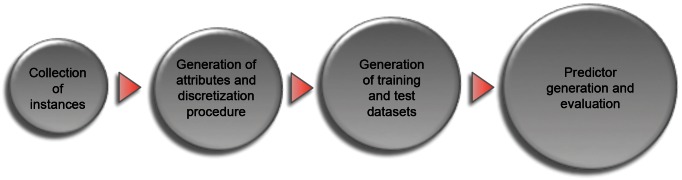
A general workflow of the procedures adopted in this work.

UNISSPI is a prediction model based on the combination of other models (see further details in the final of this section) constructed according to the typical steps performed in an ML procedure: 1 - collection of instances (in this case, known PPIs and no-PPIs) and selection of features to be used as learning attributes (in this case, a set of physicochemical features); 2 - construction of training datasets; 3 - selection of the ML algorithm to be trained (in this case, the J48 algorithm); 4 - generation and evaluation of the prediction model (Figure 5). The details of all procedures are presented in Figures S4, S5, S6, S7, S8, S9. Moreover, important definitions are presented in Text S1.

### Collection and selection of instances for the training and test sets

The PPIs and no-PPIs were obtained from the BIOGRID [47] and Negatome databases [48], respectively and their aa sequences were retrieved from the UniProt database [49] (details in Figure S4). From PPIs, we retrieved sequences from proteins associated with just one UniProt ID in BIOGRID. To avoid bias and misclassification, the PPIs and no-PPIs data were refined as follows: 1 – Only one example was chosen from redundant instances (example of redundancy: ab interaction = ba interaction, in which a and b represent proteins of one instance); and 2 - The instances classified as both PPIs and no-PPIs were also excluded.

Approximately 50% of the PPI examples for each species and all species-specific no-PPIs of eukaryotes were selected to build the training set. All other instances – instances from species with only one instance; instances with proteins from two different species; and instances from prokaryotes and viruses - were included in an independent test set (details in Figure S4).

### Generation of attributes, discretization procedure and dataset building

To build the attributes, we first calculated two different sets of physicochemical features for all proteins obtained: “frequency” and “composition”. The “frequency” set holds the percentages for each of the 20 aa in the protein: 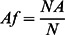, where *NA* is the number of aa of type *A* (*NA* = 1, 2, 3, …, 20), constituting a total of 20 feature descriptors. The “composition” set was obtained by grouping each aa of a protein into one of three different groups related to seven physicochemical features (details in Table S4) followed by a calculation of the percentage of each group for each feature: 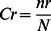, where “nr” is the number of the aa included into group 1, 2 or 3, constituting a total of 21 feature descriptors (3 groups×7 physicochemical features) (details in Figure S4). The “*N*” corresponds to the sequence length [50]. Proteins with identical features, as well as their instances in the training and test sets, were excluded from training and test datasets.

The discretization procedure was performed using the average and the standard deviation calculated for each feature descriptor for the “frequency” and “composition” sets based on the proteins in the training set (Table S5). The values of each feature descriptor in the training set were binned into four groups according to the rules described in Table 3. The protein pairs were then re-organized, and the feature descriptors of all instances that were already discretized were combined to form the final set of attributes. These procedures were conducted for all proteins for both the “frequency” and “composition” training datasets separately, resulting in the F (from “frequency”) and C (from “composition”) datasets, respectively. The symmetrical attributes (attributes of the same physicochemical features) were selected from F and C, and two other datasets were constructed, the F′ and C′ datasets, respectively. Furthermore, F and F′ were combined with C and C′, respectively, forming the F+C and F′+C′ datasets. The numbers of attributes for the F, F′, C, C′, F+C and F′+Cv training datasets were 400 (20×20 features), 20 (400−380 features), 441 (21×21 features), 21 (441−420 features), 841 (400+441 features) and 41 (841−800 features), respectively. All training sets included the same instances (details in Figure S5, S6, S7, S8).

**Table 3:** Descriptions of the bins used to discretize the attributes of the F and C datasets.

Bins	Description	Letter codes
Low	<avg−stdv	A
Moderate	>avg−stdv and < = avg	B
High	>avg and < = avg+stdv	C
Very high	>avg+stdv	D

avg, average; stdv, standard deviation.

The symmetrical attributes of all six training datasets were subjected to a standardization procedure because PPIs are supposed to be non-directional: the attributes with BA, CA, CB, DA, DB and DC were converted to AB, AC, BC, AD, BD and CD, respectively. This lack of directionality implies that f_1_p_1_ and f_1_p_2_ = f_1_p_2_ and f_1_p_1_ in a protein pair, where “f” and “p” mean “feature” and “protein”, respectively (details in Figure S9).

The “frequency” feature descriptors of all proteins from instances selected to form the test set were also subjected to the previously mentioned discretization procedures, and the average and standard deviation values obtained for the proteins of the “frequency” training set were used for the discretization (Table S5). The instances were reorganized, and the procedures for selecting the symmetrical attributes were also conducted, resulting in the F and F′ test datasets. Moreover, the symmetrical attributes of both test datasets were also subjected to the same standardization procedure as previously mentioned. All training sets included the same instances.

By the end, each training dataset (F, F′, C, C′, F+C and F′+C′) consisted of 43,365 instances from 20 eukaryotic species and 12,510 different proteins (11,609 and 944 for PPIs and no-PPIs, respectively). Moreover, each test dataset (F and F′) consisted of 42,473 instances from 78 species and 11,881 different proteins (11,576 and 305 for PPIs and no-PPIs, respectively) (Table 4–5; details in Table S6, S7).

**Table 4:** Descriptions of instances inserted in the training set.

	Number of instances		
Groups	PPI	no-PPI	Number of species per group
Amphibians	0	1	1
Annelids	0	6	3
Arthropods	6,295	1	1
Avians	9	3	1
Mammals	499	1,320	5
Plants	768	37	6
Rays	0	4	2
Yeasts	34,404	18	2
**TOTAL**	**41,975**	**1,390**	**-**

-, numbers not showed.

**Table 5:** Descriptions of instances inserted in the test set.

	Numb. of instances		Numb. of species per group
Groups	PPI	no-PPI	
*Eukaryotes	41,967	0	14
*Prokaryotes	1	168	17
*Virus	3	1	2
* **TOTAL**	**41,971**	**169**	**-**
**Eukaryote viruses and eukaryote non-host	0	13	14
**Eukaryote parasites and host	21	37	17
**Eukaryotes	15	176	34
**Eukaryotes and prokaryotes	31	13	16
**Prokaryote viruses and host	0	1	2
**Prokaryotes	0	26	8
** **TOTAL**	**67**	**266**	**-**
**TOTAL**	**42,038**	**435**	**-**

numb, numbers.

* , indicates PPIs and no-PPIs within the same species.

** , indicates PPIs and no-PPIs among different species.

-, numbers not showed.

### Balancing the training datasets and building the Random training dataset

Because the number of PPIs is much higher than no-PPIs and it is known that data imbalance degrades the performance of ML algorithms [51], it was necessary to build balanced datasets from the six original training datasets. For this purpose, 31 balanced datasets were constructed, 30 containing 2,780 instances (all 1,390 no-PPIs and 1,390 PPIs) and 1 containing 550 instances (275 no-PPIs and 275 PPIs). Before the construction of these balanced datasets, the PPI examples were randomized in an attempt to avoid bias in the datasets, and the PPIs were randomly selected from the total set of PPIs for all balanced dataset constructions. All balanced datasets for all six training datasets were named Normal datasets. From these Normal datasets, we constructed Random datasets by shuffling their classes to check whether the generated model on non-shuffled datasets learned the traits actually associated with PPIs instead of traits associated with any random subset of protein pairs. Thus, we generated 62 datasets for each of the six balanced training datasets, half of which were Normal and half were Random datasets (details in Figure S9).

### Predictive performance: validation and application tests

The prediction models were generated by training ML algorithm J48 [52] with bootstrap aggregating (bagging) [53] on all balanced datasets (Normal and Random datasets) for each group of the six training datasets. Before constructing the UNISPPI per se (a model based on the combination of 31 F′ Normal models), all generated models were first validated by estimating their predictive performances using the 10-fold cross-validation test via WEKA (Waikato Environment for Knowledge Analysis) software version 3.7.5 [54] (details in Figure S9). The K-fold cross-validation is helpful to overcome some link prediction problems [1].

To test the ability of UNISPPI to classify unseen instances, we performed an application test. Four combined models were obtained from the 31 models generated by the ML training using the F and F′ Normal and Random training datasets separately. These combined models were then used to classify the F and F′ test datasets (Table 5; Table S6, S7; procedure detailed in Text S1). Comparing the ability to classify unseen instances using models from normal and random datasets is useful for verifying the predictive ability. The same procedures were performed for the instances used in the F and F′ training datasets (Table 4; Table S6, S7). No instances in the test set were previously exposed to the J48 algorithm.

### Performance measures and statistical analysis

The estimated predictive performance of the prediction models was measured in terms of their precision, recall (equivalent to sensitivity or true positive rate) and area under the receiver operating characteristic curve (AUC). The performance measures estimated for models constructed from Normal datasets were compared using the non-parametric Mann-Whitney U statistical test [55]. In the application test, instances with a classification score of 0.50 were not classified neither PPIs nor no-PPIs.

## Supporting Information

Text S1 **Definitions and the instruction to apply UNISPPI.**
(PDF)

Figure S1 **Predictive performance of the machine learning using the Random training datasets.** The letters F, C and F+C indicate that the Random training datasets originated from the feature descriptors “frequency”, “composition”, and “frequency” plus “composition”, respectively. The prime symbol indicates the Random training datasets formed using the symmetrical attributes of the previously mentioned datasets (details in the “Material and Methods” section).
(TIF)

Figure S2 **Graphics of classification of the training sets using the F and F′ Normal combined models.** The score represents the classification of the instances as PPI. The instances were classified as PPIs or no-PPIs, and no-PPIs classification scores were converted to interaction scores. “A”, classification of instances used in the F Normal training set using the F Normal combined model; “B”, classification of instances used in the F′ Normal training set using the F′ Normal combined model; Red, PPI instances; Gray, no-PPI instances.
(TIF)

Figure S3 **Classification of the training and test sets using the F and F′ Random combined models.** The score represents the classification of the instances as PPI. The instances were classified as PPIs or no-PPIs, and no-PPIs classification scores were converted to interaction scores. “A” and “C”, classification of F test and Normal training set, respectively, using the F Random combined model; “B” and “D”, classification of F′ test and Normal training set using the F′ Normal combined model; Red, PPI instances; Gray, no-PPI instances.
(TIF)

Figure S4 **Details of the collection of instances and generation of attributes.** P, stands for proteins and the colors are relative to different proteins. The different geometrical formats represent different amino acids. “AA” and “CC”, feature descriptors.
(TIF)

Figure S5 **Generation of attributes, discretization procedure and the generation of F and C training datasets.** P stands for proteins and the colors are relative to different proteins. AVG, average; STDV, standard deviation. “AA” and “CC”, feature descriptors.
(TIF)

Figure S6 **Details of generation of F′ and C′ training datasets.** P stands for proteins and the colors are relative to different proteins. “AA” and “CC”, feature descriptors.
(TIF)

Figure S7 **Details of generation of F+C training dataset.** P stands for proteins and the colors are relative to different proteins. “AA” and “CC”, feature descriptors.
(TIF)

Figure S8 **Details of generation of F′+C′ dataset.** P stands for proteins and the colors are relative to different proteins. “AA” and “CC”, feature descriptors.
(TIF)

Figure S9 **Final building of training Normal and Random datasets; machine learning procedure and test of classification.**
(TIF)

Table S1 **Decision trees generated during the ML training.** The aa at the top of each box is relative to the attributes specified in the first and second levels of the trees. A, B, C and D indicate the low, moderate, high and very high bins, respectively (for more details, see Table 3 and the “Materials and Methods” section). Green boxes, a combination that classifies an instance as a PPI; Red boxes, a combination that classifies an instance as a no-PPI; Numbers in white boxes, a combination for which an instance can not be classified, requiring classification at the next level of the tree.
(XLS)

Table S2 **Details of the PPIs and no-PPIs correctly classified using the F′ Normal combined model.**
(XLS)

Table S3 **Comparative table of the different computational PPI methods.** Dashed lines, indicate that the underlined columns are related each other; -, indicates absent information, not showed, not used or not obviously reported in the paper.
(XLS)

Table S4 **Description of the physicochemical features in the “composition” feature descriptors.**
(XLS)

Table S5 **Average and standard deviation values from the “frequency” feature training dataset.** See “Material and Method” section and the Supplementary Text S1 to apply in your study case.
(XLS)

Table S6 **Description of amount of instances per species used in the training and test datasets.**
(XLS)

Table S7 **Description of the instances presented in the training and test datasets.** Include their UniProt accession numbers and species descriptions.
(XLS)

Dataset S1 **The model F_line_Normal_combined_model (binary file).**
(ZIP)
