# Supplementary material for: The Development of a Universal In Silico Predictor of Protein-Protein Interactions
Source: PLoS One. 2013 May 31;8(5):e65587. doi: 10.1371/journal.pone.0065587 (PMC3669264; doi:10.1371/journal.pone.0065587)
Supplement: Text S1 — Definitions and the instruction to apply UNISPPI. (PDF) [file pone.0065587.s001.pdf]

Definitions.

Supervised learning, instances and attributes.

The machine learning (ML) was the method used for the developing of this work. This method is important to the computational biology because it is able to extract knowledge from complex datasets. Here we used a supervised ML algorithm for modeling the problem of protein-protein interactions; in this case, the supervised ML algorithms discover certain characteristics from training examples and generate models based on the knowledge extracted. Thus, in an ML procedure it is necessary to build a set of training examples consisting of instances with their attributes. The instances are the individual cases collected from the real data; in our case, interacting protein pairs (PPIs) and non-interacting protein pairs (no-PPIs), that represent the two classes of interest (the positive and negative examples, respectively). These instances are characterized by features, which are technically called attributes (or descriptors) [for review see 1, 2].

Protein-protein interactions definition

We consider PPIs as biologically symmetric interactions, which protein A interacts with protein B, and *vice-versa*, [3] in an undirected way.

How to apply UNISPPI?

To use UNISPPI to classify unlabeled instances - for example a set of putative interacting proteins of one species that you are interested to investigate - the first step is to compute the “frequency” feature descriptor of all amino acids, according to the formulae $Af = \frac{NA}{N}$, where *NA* is the number of aa of type *A* (*NA*=1,2,3, …, 20). The “*N*” corresponds to the sequence length.

In order to perform the discretization procedure the numerical values have to be converted to bins (see Table 3 of “Materials and Methods” section) using the average and standard deviation values obtained from the “frequency” training dataset (values available in the Table S5). The next step is to reorganize the protein pairs, combining theirs values already discretized and to select the symmetrical attributes (20 attributes) (for example see F’ Normal training dataset building, available in Figure S5-S6).

Since PPIs are supposed to be non-directional, the standardization procedure is required. Thus, the attributes BA, CA, CB, DA, DB and DC have to be converted to AB, AC, BC, AD, BD and CD, respectively (details in Figure S9). The disposition of the attributes have to be exactly the same as follow: LysLys,LeuLeu,MetMet,AsnAsn,ProPro,GlnGln,ArgArg,SerSer,ThrThr,ValVal,TrpTrp,TyrTyr,AlaAla,CysCys,AspAsp,GluGlu,PhePhe,GlyGly,HisHis,IleIle,classe

Note that the last column is called “classe” (English-class) due to models to be generated using this word. In this way, the file should have the same word in this column. Moreover, do not insert “.” at the end of that line and use all commas without space.

Furthermore, a csv file (comma separated values) extension is required. The “?” symbol needs to be inserted at the end of all instances, at the column relative to word “classe” (English-class) and convert the csv file to arff file extension using the script as follow: java -cp /weka-3-7-5/weka.jar -Xmx12000m weka.core.converters.CSVLoader INPUT.csv > OUTPUT.arff

In the script aforementioned, “-cp” indicates the path for the program WEKA and “-Xmx12000m” is the RAM available to this task (in this case, 12,000 RAM). Note that we used the version 3.7.5 of WEKA.

After, change the header of the arff file exactly to the header as follow (you have to insert all empty lines too):

```
@relation NAME_OF_FILE.arff


@attribute LysLys {BC,AB,AC,BB,CD,CC,BD,AA,DD,AD}
@attribute LeuLeu {BC,DD,BB,CC,AC,AD,AB,CD,BD,AA}
@attribute MetMet {AB,BB,CD,BD,CC,BC,AA,AC,AD,DD}
@attribute AsnAsn {AB,AC,BB,CD,CC,BC,BD,AA,DD,AD}
@attribute ProPro {CD,AB,AC,BC,BB,DD,BD,CC,AA,AD}
@attribute GlnGln {BC,BB,AA,AB,BD,CD,DD,AC,CC,AD}
@attribute ArgArg {CC,BB,BD,AD,BC,AB,CD,AC,DD,AA}
@attribute SerSer {BC,CD,BB,AA,CC,AC,BD,AB,AD,DD}
@attribute ThrThr {AB,BC,CC,BB,CD,AD,AC,BD,AA,DD}
@attribute ValVal {AB,BC,CD,BB,AD,DD,CC,BD,AC,AA}
@attribute TrpTrp {BB,BC,AD,BD,CD,AB,CC,DD,AC,AA}
@attribute TyrTyr {AC,BB,DD,BC,AB,CD,BD,AA,CC,AD}
@attribute AlaAla {BC,BB,CD,AC,DD,AB,CC,AD,BD,AA}
@attribute CysCys {DD,BC,BB,BD,CC,CD}
@attribute AspAsp {BD,AC,CC,AD,AB,BB,BC,DD,CD,AA}
@attribute GluGlu {CC,BD,BC,AD,CD,AB,BB,DD,AC,AA}
@attribute PhePhe {AA,BC,BB,AD,AB,CC,DD,BD,AC,CD}
@attribute GlyGly {CC,BC,CD,AB,BB,AC,BD,AD,DD,AA}
@attribute HisHis {BD,BC,BB,CC,CD,AC,AD,AB,DD,AA}
@attribute IleIle {AA,AB,BB,AD,BC,CC,CD,AC,BD,DD}
@attribute classe {noppi,ppi}


@data
```

The next is step is to decompress the Dataset S1 (zip file of binary file) and rename the decompressed file exactly to “F_line_Normal_combined_model”, without those quotation marks. At the end, to classify the unlabeled instances, include the model

F_line_Normal_combined_model into the same directory with the arff file (unlabeled instances) and run the next script: java -cp /weka-3-7-5/weka.jar -Xmx3000m weka.classifiers.meta.Bagging -T INPUT.arff –l F_line_Normal_combined_model –p 0 > OUTPUT

In the script aforementioned, "-cp" indicates the path for the program WEKA, "-Xmx3000m", is the RAM available to this task (in this case, 3,000 RAM), "-T" loads the dataset to be classified and "-l" loads the model F_line_Normal_combined_model. Note that we used the version 3.7.5 of WEKA. You can follow these instructions at the "Materials and Methods" sections and at the Supporting Figures.

References

1. Larrañaga P, Calvo B, Santana R, Bielza C, Galdiano J, et al. (2005) Machine learning in bioinformatics. *Brief Bioinform* 7: 56-112.

2. Witten IH, Frank E, Hall MA (2011) Data Mining: Practical Machine Learning Tools and Techniques. San Francisco: Morgan Kaufmann. 664 p.

3. Nelson DL, Cox MM (2004) Lehninger Principles of Biochemistry. New York: W.H. Freeman and Company. 1119 p.
