# Supplementary figures and images for: The Development of a Universal In Silico Predictor of Protein-Protein Interactions
Source: PLoS One. 2013 May 31;8(5):e65587. doi: 10.1371/journal.pone.0065587 (PMC3669264; doi:10.1371/journal.pone.0065587)

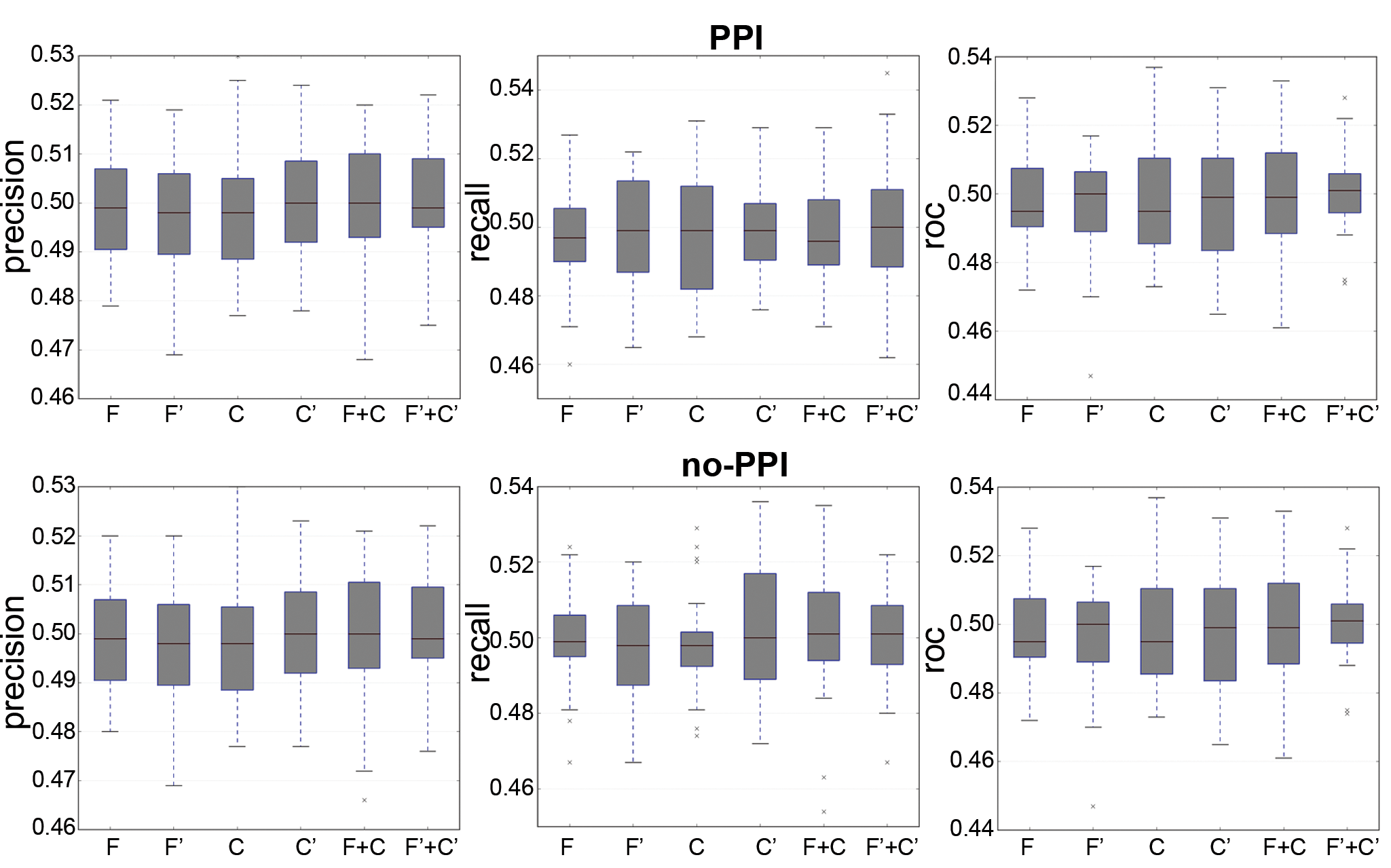

Supplement: Figure S1 — Predictive performance of the machine learning using the Random training datasets. The letters F, C and F+C indicate that the Random training datasets originated from the feature descriptors “frequency”, “composition”, and “frequency” plus “composition”, respectively. The prime symbol indicates the Random training datasets formed using the symmetrical attributes of the previously mentioned datasets (details in the “Material and Methods” section). (TIF) [file pone.0065587.s002.tif]

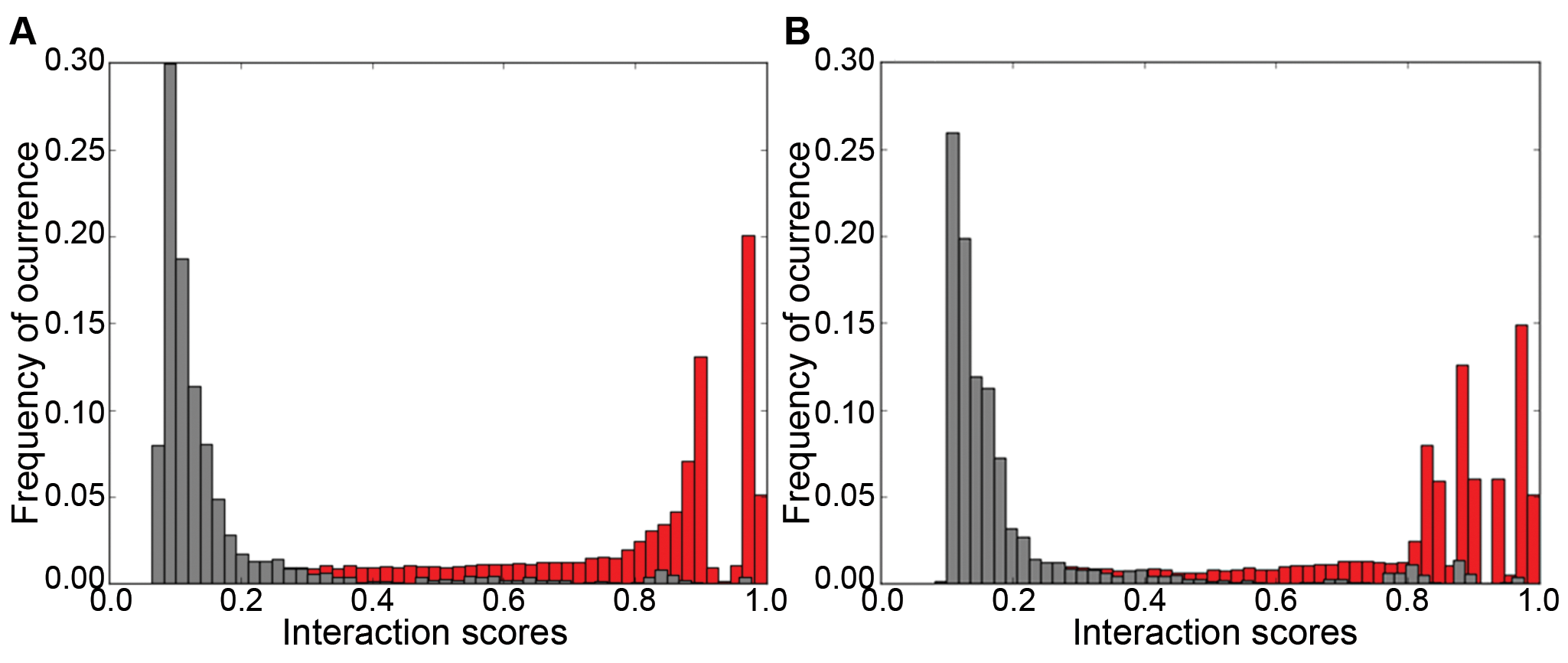

Supplement: Figure S2 — Graphics of classification of the training sets using the F and F′ Normal combined models. The score represents the classification of the instances as PPI. The instances were classified as PPIs or no-PPIs, and no-PPIs classification scores were converted to interaction scores. “A”, classification of instances used in the F Normal training set using the F Normal combined model; “B”, classification of instances used in the F′ Normal training set using the F′ Normal combined model; Red, PPI instances; Gray, no-PPI instances. (TIF) [file pone.0065587.s003.tif]

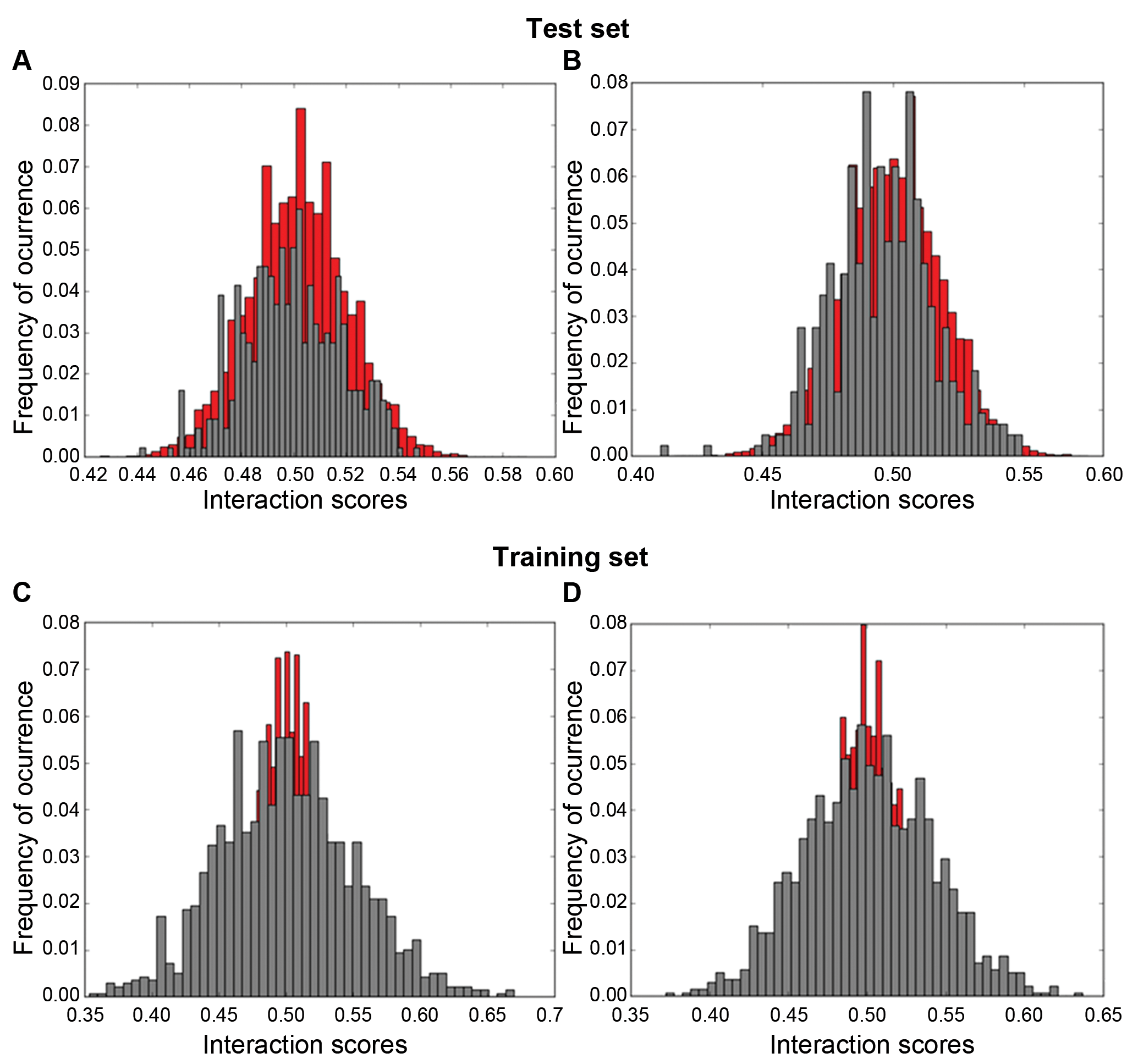

Supplement: Figure S3 — Classification of the training and test sets using the F and F′ Random combined models. The score represents the classification of the instances as PPI. The instances were classified as PPIs or no-PPIs, and no-PPIs classification scores were converted to interaction scores. “A” and “C”, classification of F test and Normal training set, respectively, using the F Random combined model; “B” and “D”, classification of F′ test and Normal training set using the F′ Normal combined model; Red, PPI instances; Gray, no-PPI instances. (TIF) [file pone.0065587.s004.tif]

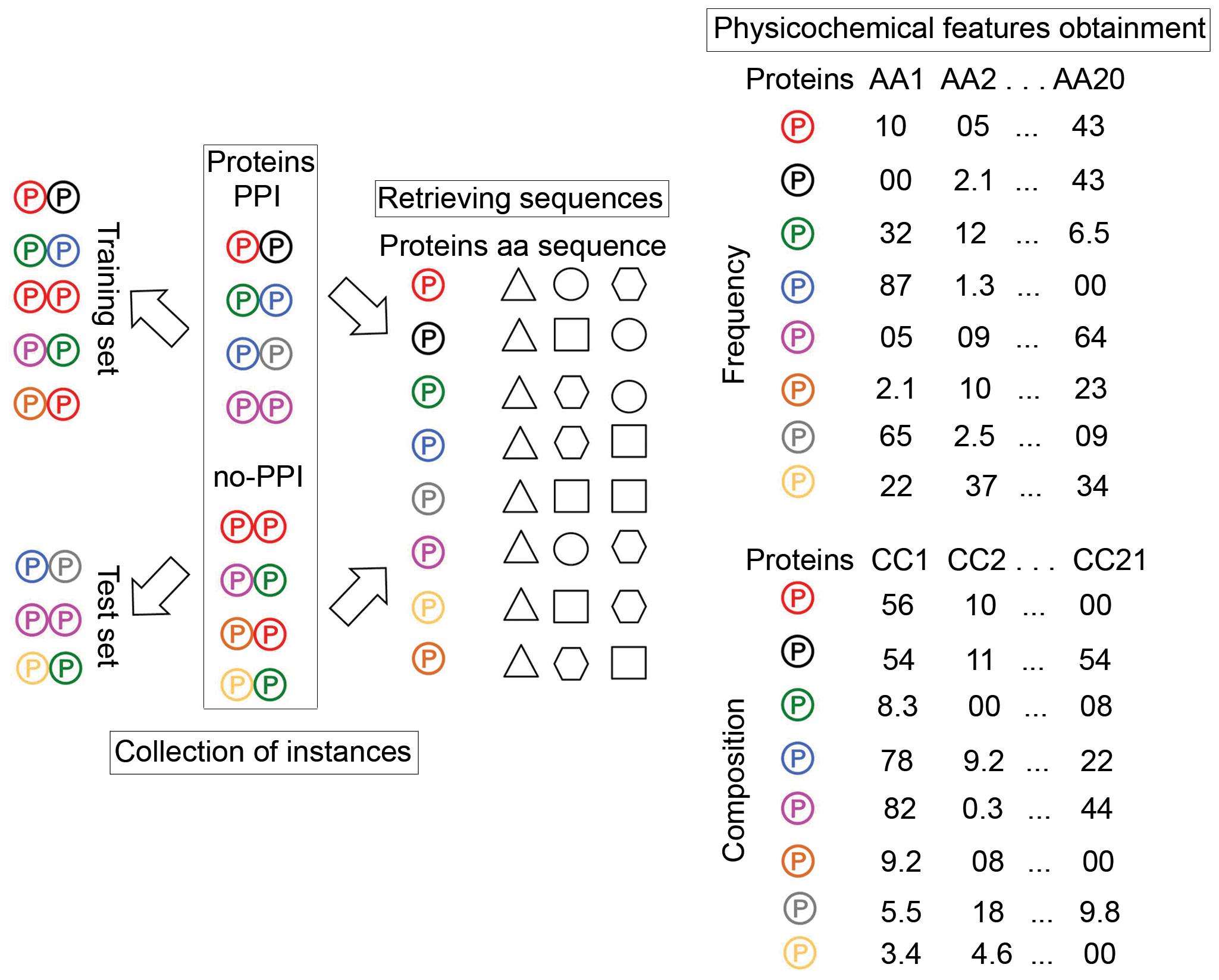

Supplement: Figure S4 — Details of the collection of instances and generation of attributes. P, stands for proteins and the colors are relative to different proteins. The different geometrical formats represent different amino acids. “AA” and “CC”, feature descriptors. (TIF) [file pone.0065587.s005.tif]

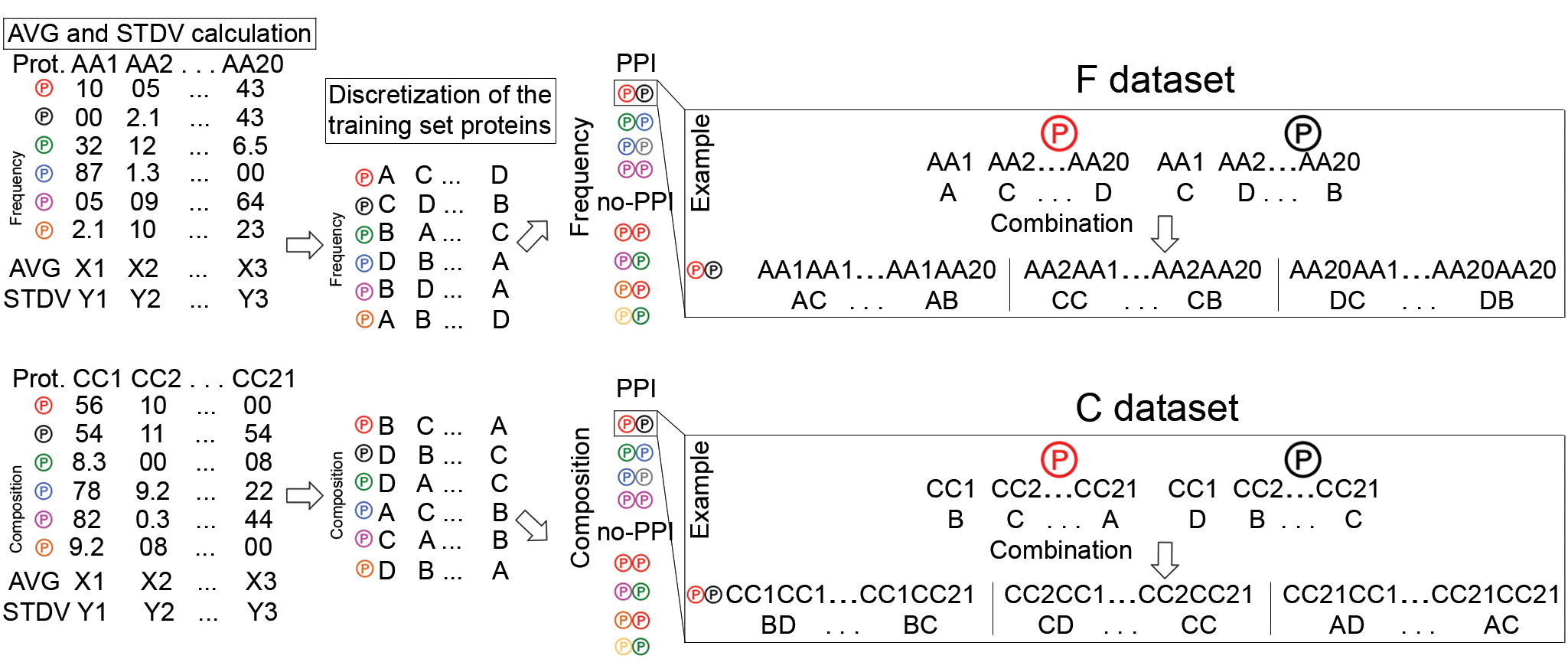

Supplement: Figure S5 — Generation of attributes, discretization procedure and the generation of F and C training datasets. P stands for proteins and the colors are relative to different proteins. AVG, average; STDV, standard deviation. “AA” and “CC”, feature descriptors. (TIF) [file pone.0065587.s006.tif]

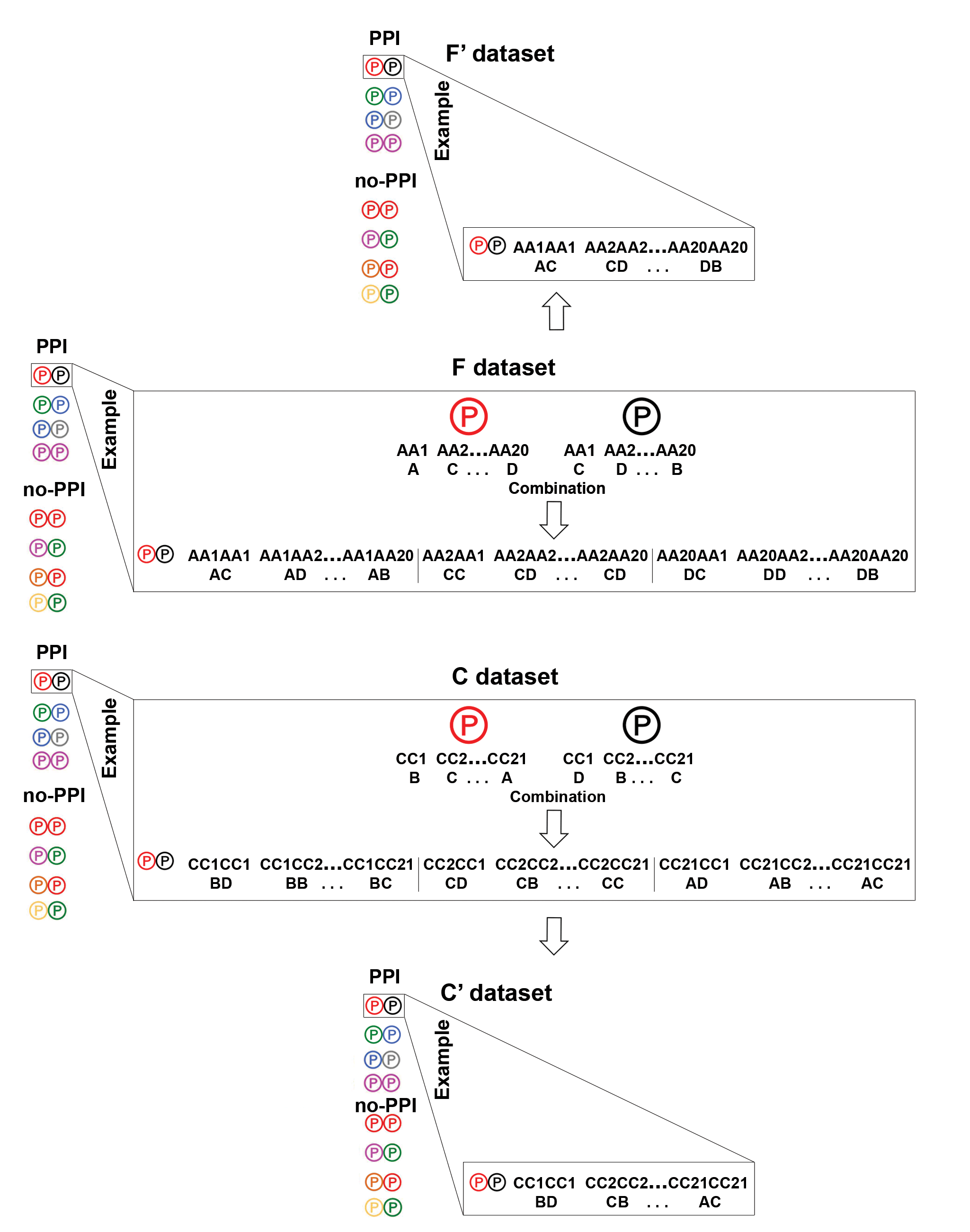

Supplement: Figure S6 — Details of generation of F′ and C′ training datasets. P stands for proteins and the colors are relative to different proteins. “AA” and “CC”, feature descriptors. (TIF) [file pone.0065587.s007.tif]

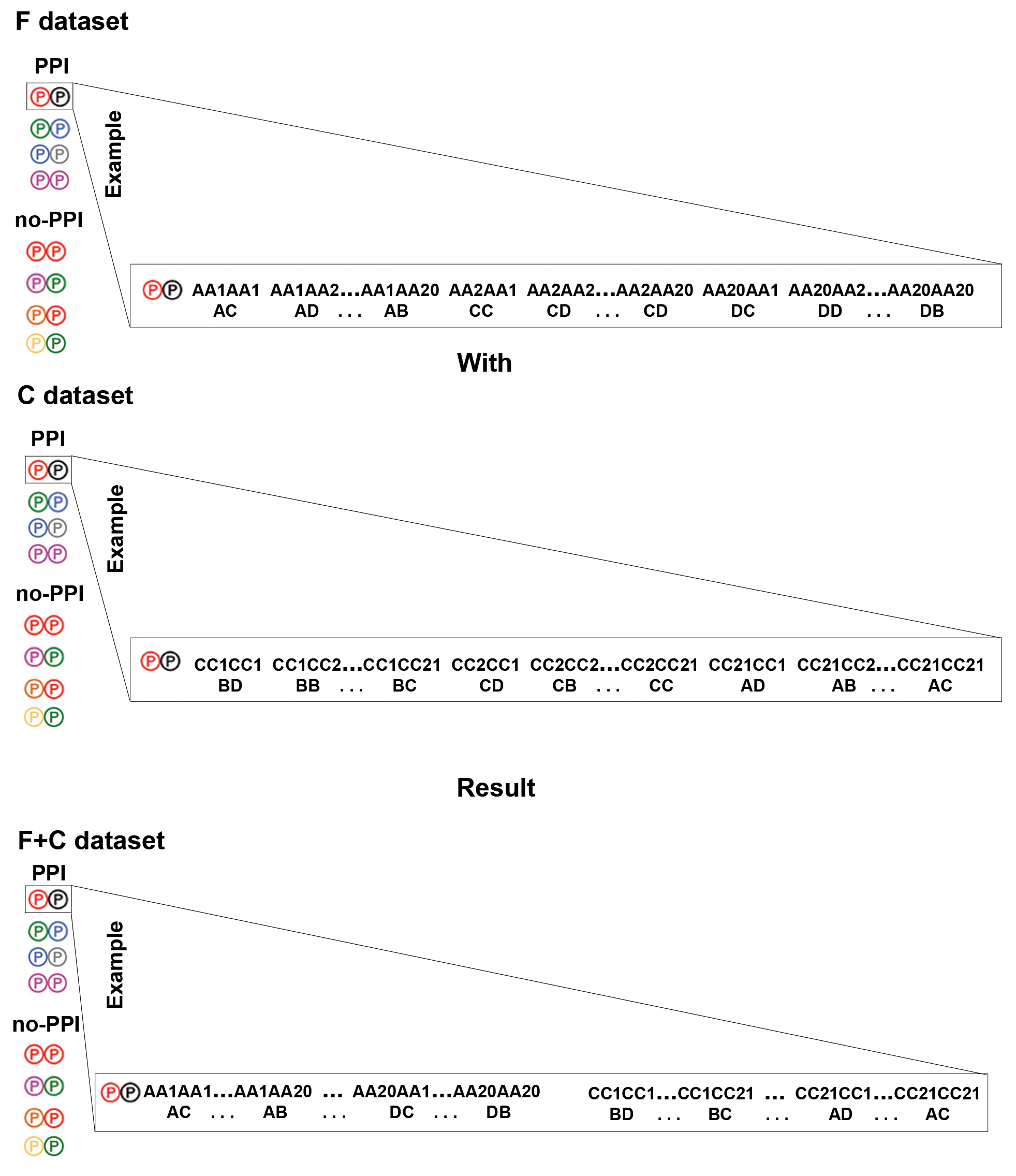

Supplement: Figure S7 — Details of generation of F+C training dataset. P stands for proteins and the colors are relative to different proteins. “AA” and “CC”, feature descriptors. (TIF) [file pone.0065587.s008.tif]

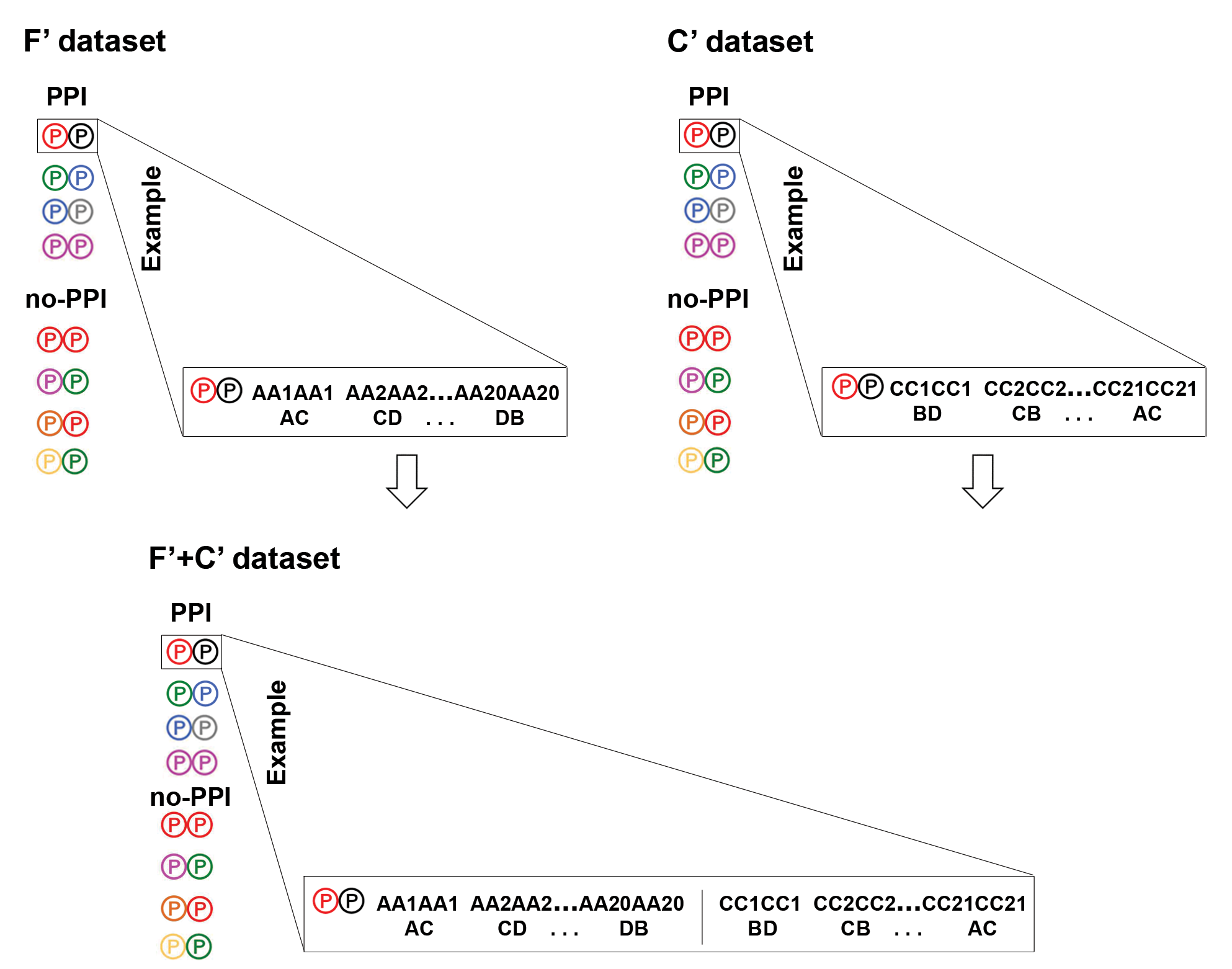

Supplement: Figure S8 — Details of generation of F′+C′ dataset. P stands for proteins and the colors are relative to different proteins. “AA” and “CC”, feature descriptors. (TIF) [file pone.0065587.s009.tif]

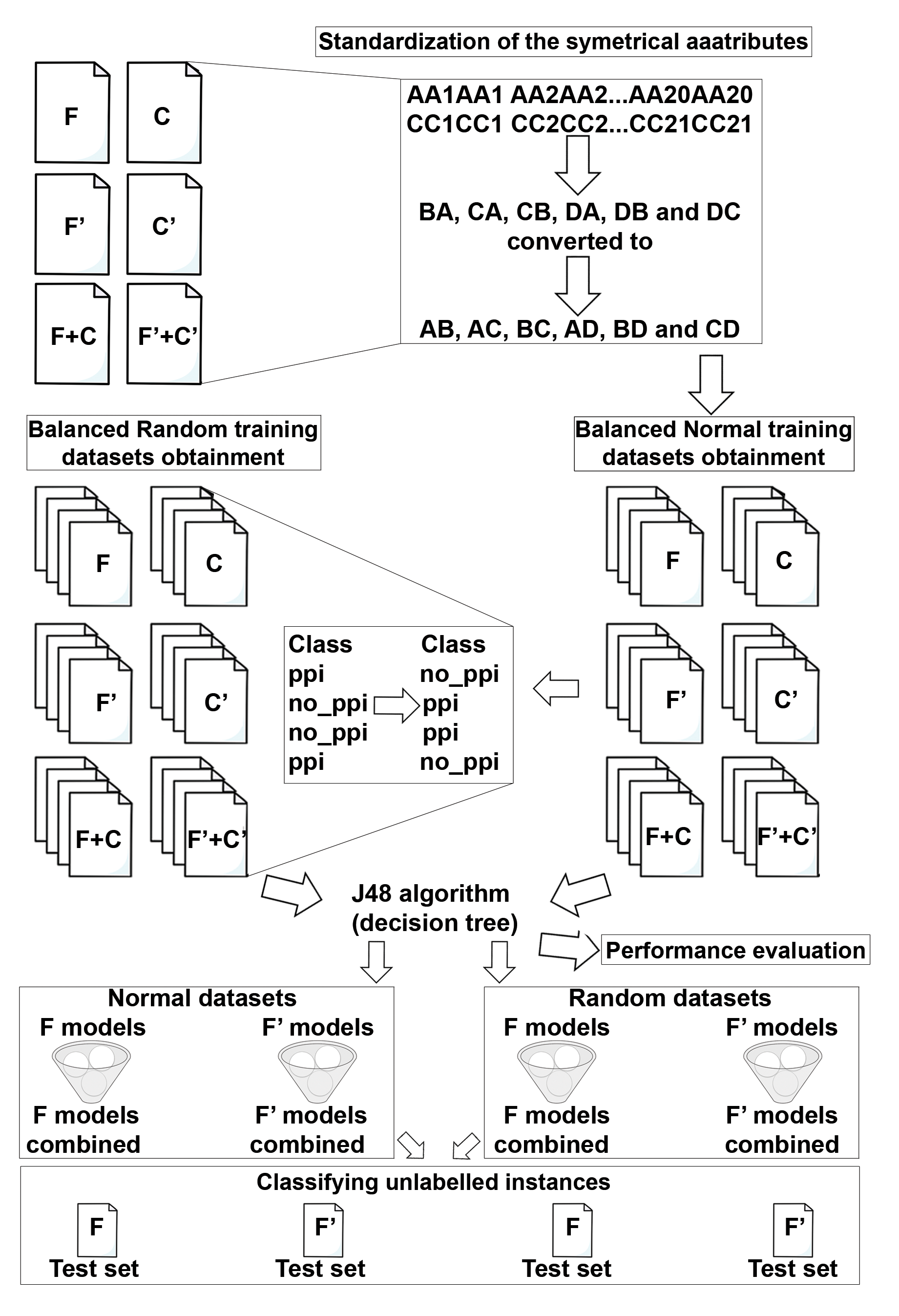

Supplement: Figure S9 — Final building of training Normal and Random datasets; machine learning procedure and test of classification. (TIF) [file pone.0065587.s010.tif]
