# Supplementary material for: The Development of a Universal In Silico Predictor of Protein-Protein Interactions
Source: PLoS One. 2013 May 31;8(5):e65587. doi: 10.1371/journal.pone.0065587 (PMC3669264; doi:10.1371/journal.pone.0065587)
Supplement: Dataset S1 — The model F_line_Normal_combined_model (binary file). (ZIP) [file pone.0065587.s018.zip › Supporting Dataset S1/README.rtf]

READMEThe file called "Supporting Dataset S1" is the model F' Normal combined model (UNISPPI per se) generated in the present work.This file is a binary file and we advice do not open that using any command line or text editor.Follow the protocol available in Supporting Text S1 to use this model for classifying unlabeled instances.
